# Toll‐like receptors, long non‐coding RNA NEAT1, and RIG‐I expression are associated with HBeAg‐positive chronic hepatitis B patients in the active phase

**DOI:** 10.1002/jcla.22886

**Published:** 2019-03-29

**Authors:** Yongbin Zeng, Wennan Wu, Ya Fu, Shanjian Chen, Tianbin Chen, Bin Yang, Qishui Ou

**Affiliations:** ^1^ Department of Laboratory Medicine The First Affiliated Hospital of Fujian Medical University Fuzhou China; ^2^ Department of Gene Diagnosis Fujian Medical University Fuzhou China

**Keywords:** active phase, chronic hepatitis B, nuclear‐enriched abundant transcript 1, retinoic acid‐inducible gene I, toll‐like receptor

## Abstract

**Background:**

Innate immunity plays a crucial role in host‐virus interactions and greatly influences viral replication including HBV infection. However, few studies have investigated the possible antiviral immune roles played by TLRs, RIG‐I, and long no‐coding RNA NEAT1 in chronic HBV infection (CHB) patients in clinical samples and their relationships among immune responses. In this study, we sought to investigate the mRNA expression levels of TLR1‐10, RIG‐I, and NEAT1 expression in HBeAg‐positive CHB treatment‐naïve patients with the active phase.

**Methods:**

The expression levels of TLR1‐10, RIG‐I, and NEAT1 of CHB patients with the active phase and healthy controls were measured by qPCR. Serum HBV DNA and routine liver biochemistry including ALT, etc were also measured to evaluate the impaired physiological function of the liver affected by CHB.

**Results:**

The expression levels of TLR1 and TLR6 in CHB with active phase were remarkably lower than that in healthy controls. The levels of TLR3 in CHB patients with active phase were remarkably higher than that in healthy controls. The total NEAT1 expression was abnormally decreased in CHB patients as compared with healthy controls. The levels of RIG‐I were significantly decreased in CHB patients in the active phase when compared to healthy controls. The expression of TLR6 and RIG‐I was closely correlated with NEAT1 expression. TLR6 level was positively correlated with RIG‐I level.

**Conclusion:**

Chronic HBV infection can alter the innate immune response by downregulating functional expression of TLR1, TLR6, NEAT1.

## 1 INTRODUCTION

Hepatitis B virus (HBV) infection is still one of the biggest public health challenges and over 650 000 people die each year due to HBV‐associated liver diseases, such as cirrhosis and hepatocellular carcinoma.^1^ Numerous evidences have shown that virological itself, host genetic background as well as immunological factors are associated with persistence of HBV infection.^2^ Among the host immunological factors, innate immunity plays a crucial role in host‐virus interactions, and greatly influences viral replication and the clinical outcome of HBV infection.^3^

Toll‐like receptors (TLRs) belong to a family of proteins named pattern recognition receptors (PRRs) and also participate in innate immunity and adaptive immunity response by recognizing invading pathogens.^4^ TLRs can recognize patterns of molecules of the antigens collectively termed as pathogen‐associated molecular patterns (PAMPs).^4^ Previous studies demonstrated that TLR ligands may suppress HBV replication in vitro and in vivo.^5^, ^6^ Activation of TLR‐mediated immunity may serve as an additional immunotherapeutic option for treating chronic HBV infection in combination with antiviral treatment.^6^ HBV may interact with the TLR signaling cascade and the change in TLRs expression might be critically important for the possible explanation for escape mechanisms of virus‐induced immune modulation.^3^

Besides, one of the key PRRs, retinoic acid‐inducible gene I (RIG‐I), has attracted much attention as its role in recognizing viral dsRNA to activate the antiviral innate responses. The process of dsRNA identification RIG‐I activates the downstream signaling pathways by affecting central adaptor protein (MAVS; also known as IPS‐1, VISA, or Cardif) and these, in turn, lead to activation of the interferon regulatory transcription factor 3 (IRF3) and nuclear factor kappa B (NF‐kB), the two important known proinflammatory transcription factors and production of type Ⅰ and type III IFNs and inflammatory cytokines to cause defected immune responses against HBV.^7^

In addition to the importance of TLRs and RIG‐I in the immune response, recently increasing evidence has also confirmed the crucial roles of long non‐coding RNAs (lncRNAs) profile in modulating host innate immunity during viral infection as well.^8^, ^9^ One of the lncRNAs, nuclear‐enriched abundant transcript 1(NEAT1), was recently known as a virus‐induced lncRNA (VINC) and was considered to be critical for the immune response against virus, however different virus infection, the different expression of NEAT1 observed.^8^, ^9^, ^10^ LncRNA NEAT1 expression is upregulated after Hantaan virus (HTNV), HIV‐1 infection, and HSV‐1 infection, whereas it is downregulated upon Zika virus infection.^8^, ^9^, ^10^, ^11^ But the roles of NEAT1 in HBV infection, especially the innate immunity of chronic HBV infection at the active phase, are currently unclear.

Considering the possible antiviral immune roles played by TLRs, RIG‐I, and NEAT1 in CHB patients and their interaction modulation of immune responses, it may be hypothesized that any alteration in expression of these molecules is capable to affect pathogenesis of CHB. Consequently, here, the aim of present study was to analyze the basal mRNA expression levels of TLR1‐10, RIG‐I, and NEAT1 expression in HBeAg‐positive CHB treatment‐naïve patients with active phase subjects.

## 2 MATERIALS AND METHODS

### 2.1 Human subjects

A total of 40 HBeAg‐positive CHB patients at the active phase (ALT ≥ 2 times the upper limit of normal) who were naive to antiviral therapy were recruited in the Center of Liver diseases of the First Affiliated Hospital of Fujian Medical University from June 2017 to October 2017. The diagnosis of CHB patients was according to Guideline of Prevention and Treatment for Chronic Hepatitis B (2015 Version), enacted by the Chinese Society of Hepatology and Chinese Society of Infectious Diseases, Chinese Medical Association. All patients had positive HBsAg for at least 6 months and were positive for HBeAg and negative for antibodies to HBeAg (HBeAb). A total of 26 healthy controls (negative for any HBV serological markers) were also included in this study. The protocol was approved by the ethics committee of The First Affiliated Hospital of Fujian Medical University and in accordance with the 1975 Declaration of Helsinki. Informed consent was also obtained. The 26 age‐ and sex‐matched HBV‐free healthy controls were collected from health examination center of the First Affiliated Hospital of Fujian Medical University tested negative for human immunodeficiency virus (HIV), hepatitis C virus (HCV), and other DNA or RNA virus.

### 2.2 Serological assays and HBV DNA assays

Routine biochemical tests including total protein (TP), albumin (Alb), alanine aminotransferase (ALT), aspartate aminotransferase (AST), total bilirubin (TBil), direct bilirubin (DB), and indirect bilirubin (IDB) were detected by automated biochemical technique (Siemens Healthcare Diagnostics, USA). Serum HBV DNA was quantified using the TaqMan polymerase chain reaction (PCR) assay (Sansure Biotech, China) on ABI 7500 Real‐Time PCR System (Life Technologies, USA), which has a detection limit of 20 IU/mL. HBsAg, HBsAb, HBeAg, HBeAb, and HBcAb were measured using a commercial chemiluminescent microparticle immunoassay kit with the Architect i4000SR System (Abbott Laboratories, USA).

### 2.3 RNA isolation, cDNA preparation, and quantitative real‐time PCR

Total RNA was isolated by using RNA extraction kit (TransGen Biotech, Beijing) following the manufacturer's instructions. The RNA was reverse‐transcribed into complementary DNA using a complementary DNA synthesis kit (TransGen Biotech, Beijing). Real‐time PCR was performed on ABI 7500 Real‐Time PCR system (Life Technologies, USA). The 25‐μl PCR amplification reaction mixtures contained 12.5 μL of 2 × SYBR Premix Ex Taq^™^ (Takara, Japan), 1.0 μL of each primer (10 μmol/L), 0.5 μL of 50 × ROXⅡreference dye (Takara, Japan), 9.0 μL of ddH_2_O, and 2.0 μL of cDNA. Real‐time PCR conditions were initial denaturation at 95°C for 30 seconds, followed by 40 cycles of denaturation at 95°C for 5 seconds, and annealing/extension at 60°C for 30 seconds. The relative quantity of the target mRNA was normalized to the level of the internal control GAPDH mRNA level. The primer pairs used to detect NEAT1, TLR1‐10, RIG‐I, and the internal control genes are listed in Table 1.

**Table 1:** Primer sequences used for real‐time RT‐PCR

Name	Sequence (5′‐3′)	Expected length
F‐TLR1	TTCACAGTGTCTGGTACACGCAT	101
R‐TLR1	ACCGTGTCTGTTAAGAGATTATTGGA	
F‐TLR2	GCCTCTCCAAGGAAGAATCC	144
R‐TLR2	TCCTGTTGTTGGACAGGTCA	
F‐TLR3	ACAACTTAGCACGGCTCTGGA	124
R‐TLR3	ACCTCAACTGGGATCTCGTCA	
F‐TLR4	AATCTAGAGCACTTGGACCTTTCC	116
R‐TLR4	GGGTTCAGGGACAGGTCTAAAGA	
F‐TLR5	CCATAGATTTTTCCTCCAACCAAATA	141
R‐TLR5	TCATACATTTTCCCCAGTCCACT	
F‐TLR6	CATCCTATTGTGAGTTTCAGGCAT	121
R‐TLR6	GCTTCATAGCACTCAATCCCAAG	
F‐TLR7	GGAGGTATTCCCACGAACACC	141
R‐TLR7	TGACCCCAGTGGAATAGGTACAC	
F‐TLR8	AAACTTGACCCAACTTCGATACCTAA	101
R‐TLR8	GATCCAGCACCTTCAGATGAGG	
F‐TLR9	GGACCTCTGGTACTGCTTCCA	151
R‐TLR9	AAGCTCGTTGTACACCCAGTCT	
F‐TLR10	AAGAAAGGTTCCCGCAGACTT	131
R‐TLR10	TGTTATGGCATAGAATCAAAACTCTCA	
F‐RIG‐I	CCATGTAAGACTTGCCTGCTT	74
R‐RIG‐I	AAGAGGCTTAATAGATTCACAGTTCC	
F‐NEAT1	CTTCCTCCCTTTAACTTATCCATTCAC	116
R‐NEAT1	CTCTTCCTCCACCATTACCAACAATAC	
F‐GAPDH	GCACCGTCAAGGCTGAGAAC	267
F‐GAPDH	TGGTGAAGACGCCAGTGGA	

### 2.4 Statistical analysis

The statistical analysis was performed using statistical analysis software SPSS version 16.0 (SPSS Inc, USA) and GraphPad Prism software version 5.0 (GraphPad Software, USA). Continuous variables are expressed as means ± standard deviations. Continuous variables were conducted using the Student's *t* test. Nonparametric statistical analysis was performed using the Mann‐Whitney *U* test for unpaired observations. The categorical variables were analyzed using the χ^2^ test. Correlations were determined using Spearman's correlation test. *P* < 0.05 was considered as statistical significance.

## 3 RESULTS

### 3.1 The characteristics of CHB patients at the active phase and healthy controls

This study consisted of 40 chronic HBV patients at the active phase and 26 healthy control subjects. The clinical background of the patients and healthy control subjects were described in Table 2. As shown in Table 2, all the patients in CHB group were HBsAg and HBV DNA positive.

**Table 2:** The characteristics of HBeAg‐positive CHB patients and healthy controls

Characteristic	CHB at active phase	Healthy controls	Statistical value	*P* value
Number	40	26	NA	NA
Age	32.51 ± 8.66	28.77 ± 6.80	4.72	0.068
Gender (M/F)	24/16	18/8	0.444	0.586^b^
Total protein	68.91 ± 1.059	72.64 ± 0.9732	2.45	0.0171^a^
Albumin	40.56 ± 0.8185	43.83 ± 0.5622	2.97	0.0043^a^
ALT	368.64 ± 483.90	17.23 ± 9.83	3.69	0.000^b^
AST	229.17 ± 324.37	19.15 ± 4.88	3.29	0.002^b^
HBsAg (log_10_ IU/mL)	3.25 ± 1.09	NA	NA	NA
HBV DNA (log_10_ IU/mL)	6.18 ± 2.05	NA	NA	NA
Total bilirubin (mg/dL)	29.69 ± 42.77	11.80 ± 3.79	2.12	0.038^b^
Direct bilirubin (mg/dL)	18.20 ± 35.58	4.60 ± 2.16	1.94	0.057^b^
Indirect Bilirubin (mg/dL)	12.03 ± 8.53	7.79 ± 2.60	2.45	0.017^b^

Values are expressed as mean ± SD.

ALT, alanine aminotransferase; AST, aspartate aminotransferase; CHB, chronic hepatitis B; NA, not applicable.

a Pearson's χ^2^.

b Student's *t* test.

Comparison with control group, the serum level of total protein (TP) and albumin (Alb) in HBV group was obviously decreased. As the total protein and albumin is mainly synthesized in liver, the decreased level of these two proteins suggesting the impaired physiological function of the liver. Furthermore, liver injury index was detected. The serum levels of alanine aminotransferase (ALT), aspartate aminotransferase (AST), and total bilirubin (TB) were significantly increased in chronic HBV patient group compared to the healthy control group, indicating the hepatocytes injury caused by CHB infection at the active phase.

### 3.2 Quantitative analysis of TLR 1‐10 mRNA expression in peripheral blood

**Figure 1:**
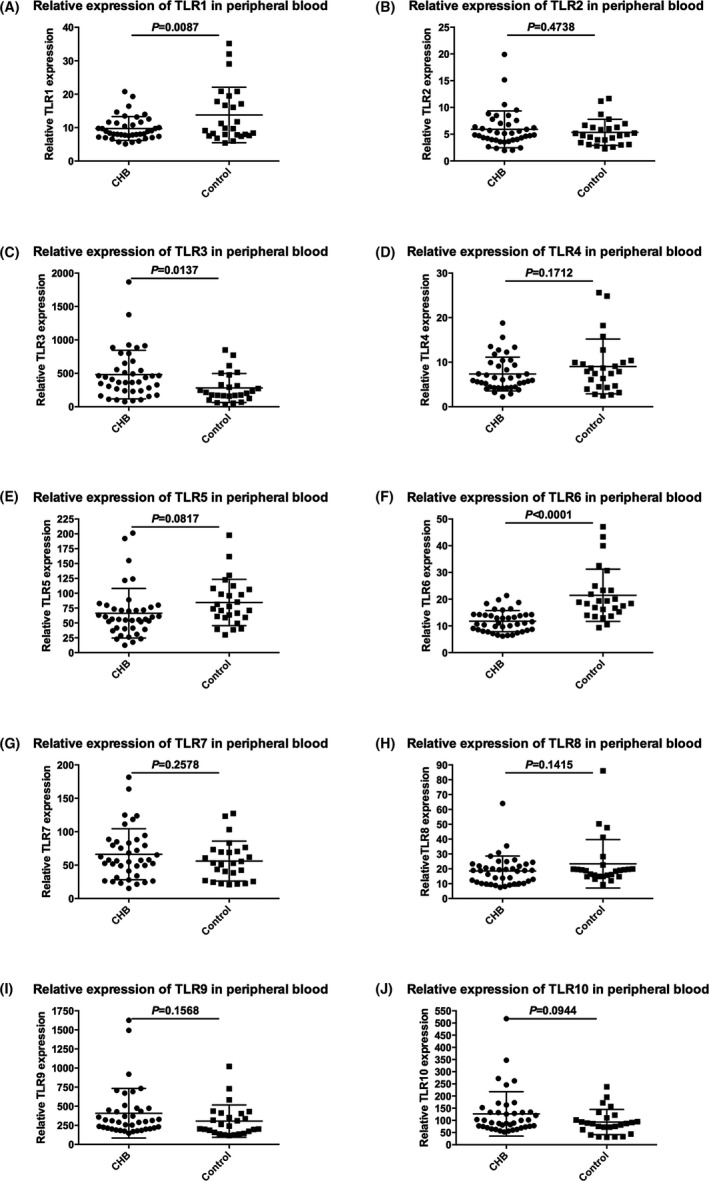
Quantitative analysis of TLR1‐10 mRNA expression from CHB patients and healthy donors

The expression levels of TLR1‐10 mRNA were measured from 40 chronic HBV‐infected patients and 26 healthy donors by quantitative real‐time PCR (Figure 1). Obviously, our results showed that expression levels of TLR1 and TLR6 in CHB with active phase was remarkably lower than that in healthy donors, 9.761 ± 0.5634 vs 13.77 ± 1.627, *t* = 2.705, *P* = 0.0087 and 11.78 ± 0.6227 vs 21.45 ± 1.916, *t* = 5.615, *P* < 0.0001, respectively. The expression levels of TLR3 in CHB with active phase were remarkably higher than that in healthy donors, 481.2 ± 57.48 vs 280.3 ± 42.53, *t* = 2.536, *P* = 0.0137. In contrast, TLR2, TLR4, TLR5, TLR7, TLR8, TLR9, and TLR10 shown no significant differences between the two groups.

### 3.3 Downregulation of NEAT1 gene expression in peripheral blood in chronic HBV infection and healthy donors

**Figure 2:**
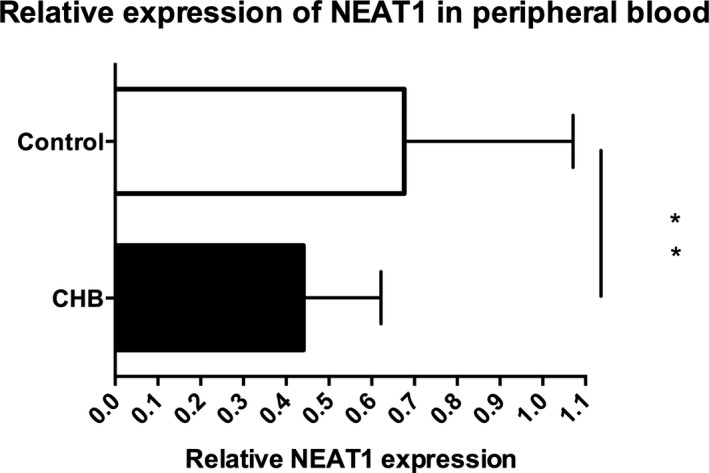
Expression of NEAT1 in CHB patients at active phase and healthy controls **P < 0.01

As shown in Figure 2, the total NEAT1 expression was abnormally decreased in CHB patients as compared with healthy donors (0.4406 ± 0.02864 vs 0.6763 ± 0.07744, *t* = 3.291, *P* = 0.0016).

### 3.4 Analysis of RIG‐I expression in peripheral blood

**Figure 3:**
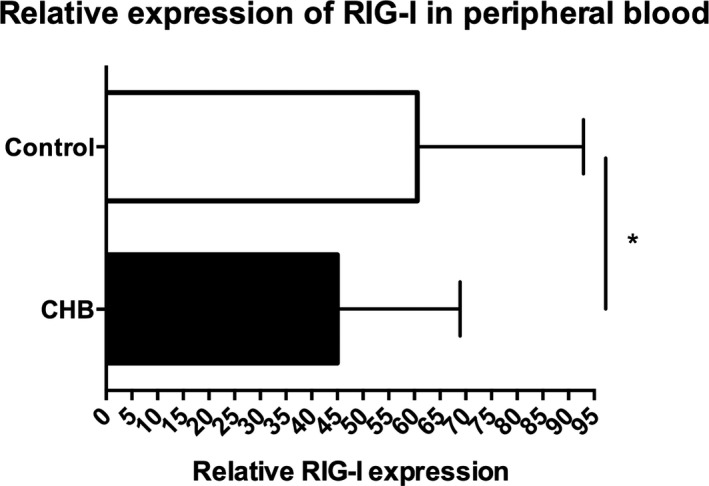
Expression of RIG‐I in CHB patients at active phase and healthy controls *P < 0.05

Figure 3 showed that RIG‐I expression was significantly decreased in CHB patients in comparison to healthy donors (45.01 ± 3.776 vs 60.55 ± 6.343, *t* = 2.244, *P* = 0.0283).

### 3.5 A positive correlation between NEAT1, TLR, and RIG‐I genes in CHB patients

To understand the possible role and status of innate immunity function during chronic HBV infection at the active phase, we further analyzed the correlation between NEAT1, TLR, and RIG‐I genes.

**Figure 4:**
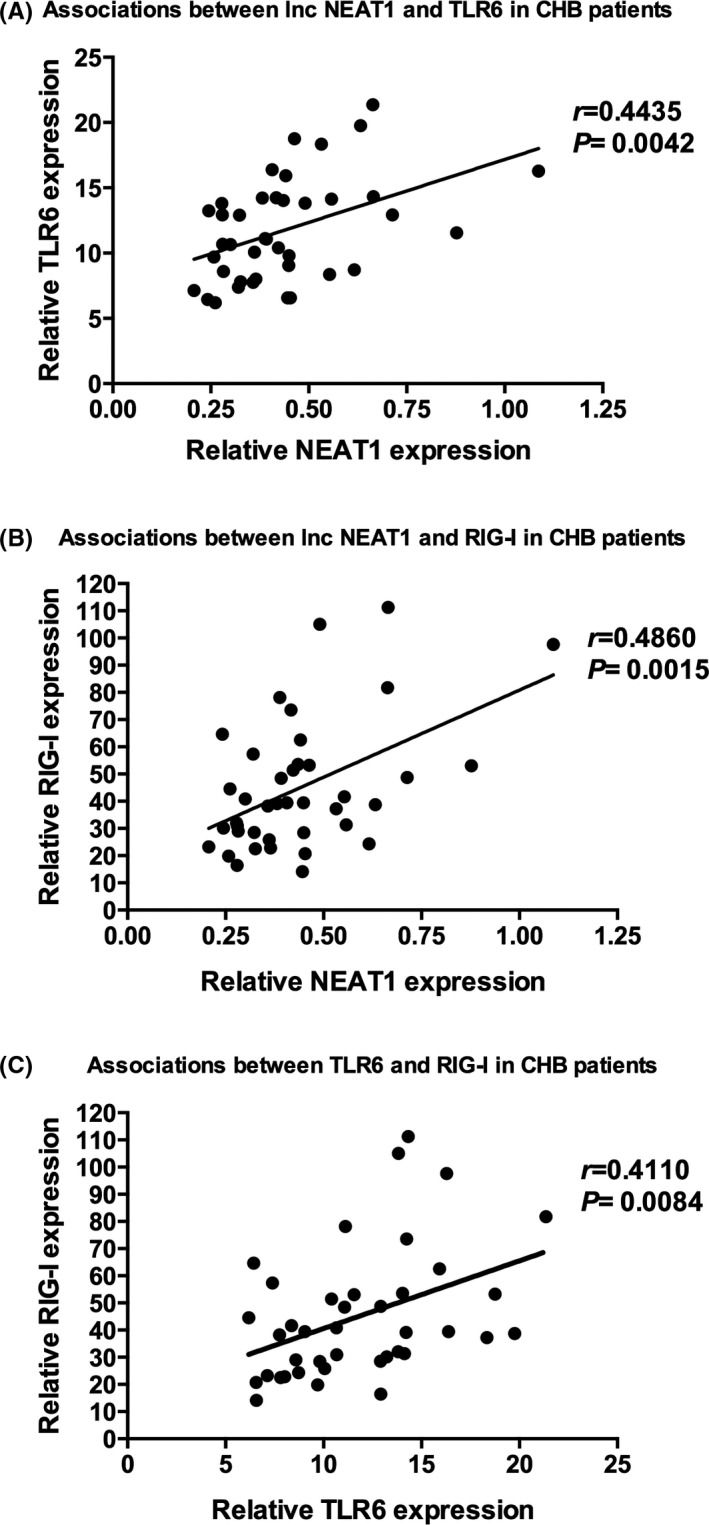
Correlation between NEAT1, TLR, and RIG‐I genes in CHB patients at active phase and healthy controls

Statistic analysis showed that the expression of TLR6 and RIG‐I was closely correlated with NEAT1 mRNA expression (*r* = 0.4435, *P* = 0.0042; *r* = 0.4860, *P* = 0.0015; Figure 4A,B). It is interesting to observe that TLR6 expression levels were positively correlated with RIG‐I levels (*r* = 0.4110, *P* = 0.0084, Figure 4C).

## 4 DISCUSSION

Toll‐like receptors (TLRs) are central players in the early host immune response to acute viral infection and have been shown to play a crucial role in defense against microorganisms.^3^ Evidences have demonstrated that the activation of TLR signaling pathway by HBV was able to inhibit HBV replication.^6^, ^12^ Thompson et al^13^ documented that TLR2 inhibited HBV replication in hepatoma cell lines in vitro. Luangsay et al^14^ found that strong antiviral activity against HBV was obtained in the presence of the ligands for TLR1/2, TLR4, and RIG‐I/MDA‐5. In the present study, using quantitative real‐time PCR, we characterized the expression levels of TLRs 1‐10 transcripts during chronic hepatitis B infection at the active phase characteristics with high ALT level (ALT ≥ 2 times the upper limit of normal). Results of the current study showed that the expression levels of TLR1, TLR6 in the peripheral blood from CHB patients were significantly decreased in comparison with healthy controls which were consistent with the reports of Chen et al^15^ to a certain extent. Nevertheless, Wang et al^3^ found that TLR1 in CHB with active phase were moderately higher compared with healthy individuals. They also found TLR2/4/6 mRNA was upregulated in active stage of CHB. The conflicting results documented by us and the other researchers might be due to the variability of CHB infection, such as different HBV genotypes infected, different stages of CHB, and different host immune status. Although the above controversial results, what we can infer without doubt is that HBV may interact with the host through the TLR signaling cascade which mediates innate immunity in viral infection. It may also to be concluded that CHB patients may be unable to express suitable levels of these signaling molecules to induce expression of several required inflammatory factors, including proinflammatory cytokines, that are required for clearance of the HBV infection. In other words, reduced expression of these molecules may help HBV to establish chronic infection. However, the mechanisms involved required further studies.

In this study, we also investigated the expression of RIG‐I in CHB patients at active phase and health controls. The lncRNA NEAT1 is encoded on chromosome 11q13.1 and has been reported to be involved in the pathogenesis of multiple types of cancer.^16^ NEAT1 also takes part in neurodegenerative diseases such as huntington's disease.^17^ Recently, lncRNA NEAT1 has been reported to serve as an important link between virus infection and innate immune system. NEAT1 is documented as a virus inducible non‐coding RNA (VINC), and changes in NEAT1 expression have been investigated in several different viral infections. However, few studies have focused on NEAT1 in patients with CHB. In the present study, we found lncRNA NEAT1 was downregulated in peripheral blood from CHB patients. The present study was, to the best of our knowledge, the first research demonstrating the altered expression of lncRNA NEAT1 in patients with chronic HBV at the active phase. The underlying mechanism needs to be elucidated. But it can speculate that NEAT1 downregulation may delay host innate immune responses and aggravated HBV replication.

Our results also demonstrated that the expression level of RIG‐I was significantly decreased in CHB patients when compared to healthy controls. Concerning the fact that RIG‐I plays important roles in viral PAMPs recognition, decreased expression of this molecule may result in impaired HBV recognition and consequently disrupted immune responses in CHB patients. This association between decreased level of RIG‐I expression and HBV infection is in line with the ability of RIG‐I binding to viral double‐stranded RNA and induce an IFN‐based antiviral immune response. Beyond that, it is also interesting to observe that there were positive correlations among TLR6, RIG‐I, and NEAT1. However, additional experiments are needed to elucidate the precise mechanisms.

It should be noted that there are several limitations in this study. First, just the active CHB patients and health donors were included in the present study. Patients with inactive CHB patients, active CHB patients, and health donors should all be considered in further study to make the conclusion more convincing. Second, the sample size was relatively small. Therefore, more samples are needed to determine the issue discussed above in future.

In conclusion, this study suggests that chronic HBV infection can alter the innate immune response by downregulation of functional expression of TLR1, TLR6, NEAT1, and RIG‐I, which may contribute to the establishment of chronic infections. Moreover, the study provides the first experimental evidence, demonstrating that the expressions of toll‐like receptors, long non‐coding RNA NEAT1 and RIG‐I are associated with chronic HBV infection in the active phase.

## CONFLICT OF INTEREST

The authors declare that they have no conflicts of interest regarding the publication of this article.

Employment or leadership: None declared.

Honorarium: None declared.
